# Heightened risk of canine chocolate exposure at Christmas and Easter

**DOI:** 10.1136/vr.104762

**Published:** 2017-12-20

**Authors:** Peter-John M Noble, Jenny Newman, Alison M Wyatt, Alan D Radford, Philip H Jones

**Affiliations:** 1 Small Animal Teaching Hospital, University of Liverpool, Institute of Veterinary Science, Neston, UK; 2 Department of Epidemiology and Population Health, University of Liverpool, Institute of Infection and Global Health, Neston, UK; 3 University of Liverpool, Institute of Veterinary Science, Neston, UK; 4 Department of Infection Biology, University of Liverpool, Institute of Infection and Global Health, Neston, UK

**Keywords:** dogs, chocolate, poisoning

Chocolate has long been recognised as, and remains, a common cause of intoxication in dogs accounting for 25 per cent of acute presentations for intoxication.^1 2^ Case numbers in Europe and the UK have been reviewed, mostly based on reports to poisons centres.^3–6^ Chocolate toxicity results from the methylxanthine theobromine present in cocoa bean products, causing gastrointestinal (eg, vomiting), cardiovascular (eg, tachycardia) and central nervous (eg, agitation and seizure) signs.^7^ Chocolate intoxication is mostly seen in dogs^6 8^ and theobromine dose calculations based on the source of chocolate are well documented^7 9^ and available online. 10 11 The current study reviews cases of chocolate exposure presented to a large sentinel network of UK veterinary practices between 2012 and 2017.

Electronic health records were collected from 229 UK veterinary practices (500 premises) by the Small Animal Veterinary Surveillance Network (SAVSNET).^12^ Records included the consultation time, species, breed, sex and clinical free text (narrative) in which inadvertent personal identifying data contained in narratives had been redacted using deidentification software (Newman). Narratives were screened using a regular expression^13^ to identify the presence of the word ‘chocolate’, including a range of misspellings and contractions (eg, ‘choc’, ‘choclat’). Cases were tagged for study inclusion if on reading they matched a definition of potential chocolate exposure whereby ingestion triggered either specific treatment or a plan of monitoring for clinical signs of theobromine toxicity.

Data were wrangled using the Pandas library^14^ in Python V.3.4^15^ and analysed using Stata V.12.^16^ Five unmatched controls were randomly selected for each case using animals not identified by the initial screening for chocolate. To analyse risk periods, a categorical variable was generated to indicate whether a consultation occurred from one week before to two weeks after Christmas, Easter, Valentine’s Day or Halloween. Univariable analysis of the effect of sex-neutering status, age category (0 to <4 years, 4 to <8 years, 8 years and over) and risk period was undertaken. Variables that showed a significant effect were included in a multivariable logistic regression model. The effect of breed was analysed using Fisher’s exact tests for each breed separately (to accommodate small numbers of individuals involved) and interpreting the P value following a Bonferroni adjustment.

**FIG 1:**
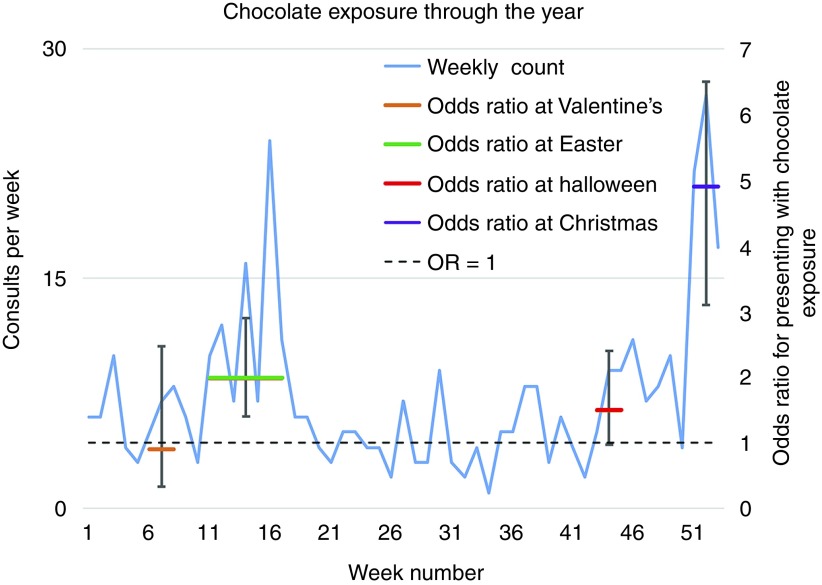
Weekly count of chocolate exposure consultations (blue line, left axis) with odds ratio for presenting with chocolate intoxication during given celebration periods (bars±95% CI, right axis). Note: Easter occurs in different weeks each year.

In total, 1722 consultations referring to chocolate were identified from 2.7 million narratives collected between November 2012 and May 2017 and of these 386 (22 per cent) narratives from 375 individual animals were identified as matching the chocolate exposure case definition. Where an animal appeared more than once, only the first consultation was retained. Many cases (101, 26 per cent) presented within one hour of ingesting chocolate and the majority (217, 56 per cent) presented within six hours. Vomiting following ingestion was frequently noted (64 cases, 17 per cent), while neurological signs (agitation, restlessness) were uncommon (12 cases, 3 per cent). Seizures were not reported in any cases. Heart rate greater than 120 bpm was noted in 28 cases (7.5 per cent). None of the clinical signs seen was considered life-threatening. Age category and risk period showed significant univariable effects and were included in a multivariable model. Chocolate exposure was significantly less common in old dogs (odds ratio (OR)=0.42, 95 per cent confidence interval (CI) 0.31 to 0.56, P<0.001) and potentially less common in middle-aged dogs (OR=0.77, CI 0.59 to 1.01, P=0.058) than dogs under four years of age. No breed was associated with an increased risk. Chocolate exposure was more commonly recorded at Christmas (OR=4.74, CI 3.04 to 7.40, P<0.001) and Easter (OR=1.97, CI 1.36 to 2.86, P<0.001) in this population than at non-festival dates (Fig 1); the Valentine’s Day and Halloween ORs were not significantly different from 1.0 (OR=0.94, CI  0.56 to 1.57, P=0.803 and OR=1.55, CI 0.97 to 2.46, P=0.065, respectively). Sources of chocolate included bars and boxes (often gift selections) of chocolate (35 cases), Easter eggs (31 cases), chocolate cake (22 cases), liqueurs (5 cases), chocolate rabbits, Santa Claus figurines, Advent calendars and Christmas tree decorations (10 cases), as well as one case involving a hot chocolate drink. Dogs ingested chocolate oranges (15 cases) and Toblerone (6 cases) and in one case, both (six of each). Chocolate co-ingestion alongside raisins (three cases) and single cases of paracetamol, ibuprofen, xylitol and onion were noted without evidence that these caused disease. While chocolate doses were often small, exceptions included ingestion of a garden of Easter eggs hidden for a large party of children. Documented treatments included activated charcoal (121 cases), apomorphine (114 cases), intravenous fluid therapy (12 cases) and anti-emetics (31 cases, usually following apomorphine). Activated charcoal therapy often followed induced emesis but in 65 cases, it was the sole therapy. Theobromine dose was noted or could be inferred from chocolate dose details in 185 narratives of which 75 narratives (41 per cent) reflected non-toxic doses (less than 20 mg/kg).^17^ In 34 of these cases, patients were still given apomorphine as a safety measure.

Here the authors describe significant peaks of chocolate intoxication, most notably at Christmas and to a lesser extent Easter, presumably reflecting the enhanced availability of seasonally-related chocolate such as Easter eggs, chocolate Santa Claus figurines and Christmas tree decorations, possibly in the hands of younger members of society. Peaks of chocolate exposure around Valentine’s day and Halloween described in German and US reviews^4 6^ were not seen in the UK, perhaps reflecting alternative romantic gift choices (or more fastidious curation by their recipient) and different festival priorities although data to support this conjecture were not available. Use of apomorphine in cases where theobromine dose appeared to be non-toxic probably reflects a belief that the low risk of toxicity in these cases outweighs that of emesis but runs contrary to recent recommendations which point out the lack of evidence for use of emetics in these cases.^17^

Chocolate ingestion has a unique seasonal pattern which merits highlighting this risk to clients, particularly in the run-up to Christmas and Easter as chocolate becomes more accessible within the household. Given the frequent use of emetics in animals with documented non-toxic doses of theobromine, further research into the risks and consequences of emetic therapy is indicated.
